# Modeling Flaw Pulse-Echo Signals in Cylindrical Components Using an Ultrasonic Line-Focused Transducer with Consideration of Wave Mode Conversion

**DOI:** 10.3390/s19122744

**Published:** 2019-06-18

**Authors:** Weixin Wang, Xiling Liu, Xiongbing Li, Gang Xu, Shuzeng Zhang

**Affiliations:** 1 School of Traffic and Transportation Engineering, Central South University, Changsha 410075, China; wwx2017@csu.edu.cn (W.W.); xlliu@csu.edu.cn (X.L.); lixb_ex@163.com (X.L.);; 2 China Rolling Stock Corporation Qingdao Sifang Co., Ltd., Qingdao 266111, China; 15265211573@163.com; 3 Key Laboratory of Traffic Safety on Track, Ministry of Education, Changsha 410075, China

**Keywords:** ultrasonic measurement model, line focused transducer, multi-Gaussian beam model, wave mode conversion, cylindrical component

## Abstract

Investigations on flaw responses can benefit the nondestructive testing of cylinders using line-focused transducers. In this work, the system function, the wave beam model, and a flaw scattering model are combined to develop an ultrasonic measurement model for line-focused transducers to predict flaw responses in cylindrical components. The system function is characterized using reference signals by developing an acoustic transfer function for line-focused transducers, which works at different distances for both planar and curved surfaces. The wave beams in cylindrical components are modeled using a multi-Gaussian beam model, where the effects of wave mode conversion and curvatures of cylinders are considered. Simulation results of wave beams are provided to analyze their propagation behaviors. The proposed ultrasonic measurement model is certified from good agreement between the experimental and predicted signals of side-drilled holes. This work provides guidance for evaluating the detection ability of line-focused transducers in cylindrical component testing applications.

## 1. Introduction

Cylindrical components have been widely used in practical applications, such as subsea pipelines and train axles [1]. These components bear complicated loadings during their service, and if there are flaws in them, their service security will be threatened. Therefore, it is important to detect defects in these components to ensure their safety in service. Ultrasonic testing provides an effective technique to nondestructively test and evaluate material properties [2,3]. When a cylindrical component is tested using the ultrasonic method, line-focused transducers can concentrate acoustic energy in a line through the cylindrical interface of the component, which is helpful for detecting flaws. In addition, such transducers have a higher sensitivity and signal-to-noise ratio than planar transducers do, while having a larger scanning area than point-focused transducers have [4]. Moreover, the equipment for the nondestructive testing method using line-focused transducers is much simpler than that using a phased array [5]. Therefore, line-focused transducers are suitable for nondestructively testing cylindrical components. Understanding the wave generation and propagation process and analyzing the properties of flaw signals are helpful to guide practical applications using line focused transducers, thus it is essential to theoretically and experimentally investigate their testing ability.

The ultrasonic measurement model (UMM) is one of the most important techniques in ultrasonic nondestructive evaluation [6]. An UMM includes the configuration of the transducer and component being inspected, as well as a description of the wave generation, propagation and reception, and it is used to predict the output signals from anomalies in the measured component [6]. Development of an UMM for line-focused transducers can benefit the applications using line-focused transducers in detection of cylindrical components: The calibration of the inspection system using a line-focused transducer is helpful to test performance parameters of such a transducer. In addition, the simulation results based on a reliable model can provide guidance in the design and optimization of efficient testing configurations.

The UMM has been widely studied in recent years. Auld first introduced the reciprocity relation to the ultrasonic nondestructive test system and proposed a basic model to make the connection between the measurement output and flaw response [7]. Based on Auld’s model, Thompson and Gray developed a general measurement model by using a quasi-plane wave to predict the received signals from relatively small flaws [8]. Later, Schmerr proposed a more complete model to predict the signals from large flaws by considering the wave field distributions over the flaw surface [9]. Recently, the development of the UMM is mainly to improve the efficiency by introducing the multi-Gaussian beam (MGB) model for planar or point-focused transducers [10,11,12,13,14]. However, since the wave beams generated by line-focused transducers are different from those of the abovementioned transducers, the modeling method cannot be directly applied when a line-focused transducer is introduced.

The flaw scattering model plays an important role in the development of the UMM, and recent studies have proposed more accurate flaw scattering models [15,16,17]. There are some standard reference reflectors, such as plat bottom hole, spherical void, and side-drilled hole (SDH) [9]. In this research, an SDH is very propitious to calibrate a line-focused transducer as most of the wave beam energy can be concentrated at the flaw surface. In the practical application of ultrasonic nondestructive evaluation, the detection method using the longitudinal wave and shear wave with oblique incident are complementary in many respects. For example, the longitudinal waves in normal incidence are sensitive to the flaws whose surface is parallel to the interface, while mode-converted shear wave inspections may improve the detectability of defects elongated perpendicular to the surface [18]. Since an SDH can well reflect the incident wave from any direction, its scattering characteristics are suitable to investigate the effects of wave mode conversion to accurately detect the defects in cylindrical components using a line-focused transducer.

In this work, an UMM using line-focused transducers is developed to predict the flaw responses from SDHs in cylindrical components. A method is proposed to calibrate the system functions with line-focused transducers. The MGB model is introduced to simulate the wave beams propagating in cylindrical components, and the effects of both cylindrical curvatures and wave mode conversion are taken into account. Simulation results are provided to analyze the wave propagation properties in cylindrical components. Experiments are conducted to validate the proposed model and check the detection sensitivity using line-focused transducers.

## 2. Theory

### 2.1. An UMM for Predicting Responses from an SDH

**Figure 1:**
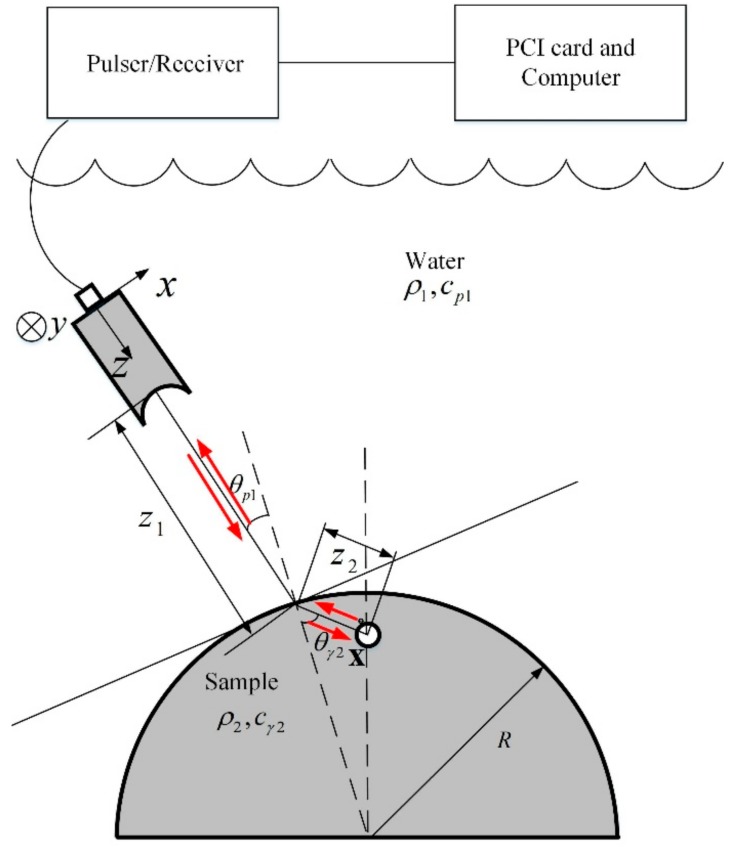
Schematic diagram of the experimental test system.

The ultrasonic test system is shown in Figure 1, in which a line-focused transducer is used to detect the SDH in a cylindrical component of radius *R* through the pulse-echo immersion method. A pulser/receiver drives the transducer to generate longitudinal waves in water. These waves will propagate to the flaw surface through the water/component interface, be reflected by the flaw and propagate back to the transducer. The wave signals are received by a PCI card and saved in an industrial computer.

Based on the reciprocity principle, the frequency components of the received voltage, $V_{0}\left(\omega \right)$, can be expressed in terms of the velocity and stress fields on the surface of the scatterer [6] as

(1) $$V_{0}\left(\omega \right)=\frac{\beta \left(\omega \right)}{\rho_{1}c_{p1}S_{R}v_{T}\left(\omega \right)v_{R}\left(\omega \right)}\cdot \int_{S_{f}}\left[\tau_{ji}^{\left(a\right)}\left(x,\omega \right)v_{i}^{\left(b\right)}\left(x,\omega \right)-\tau_{ji}^{\left(b\right)}\left(x,\omega \right)v_{i}^{\left(a\right)}\left(x,\omega \right)\right]n_{j}\left(x\right)dS$$

where $\beta \left(\omega \right)$ is the system function, which is used to describe the conversion relationship between the electricity and ultrasonic acoustics, $\rho_{1}$ and $c_{p1}$ are density and longitudinal wave velocity in water, respectively, $v_{T}\left(\omega \right)$ and $v_{R}\left(\omega \right)$ represent the average velocity fields at surfaces of the transmitter and the receiver, respectively, and $S_{R}$ is the area of the receiver. In the integration, $\tau_{ji}^{\left(m\right)}\left(x,\omega \right)$ and $v_{i}^{\left(m\right)}\left(x,\omega \right)$ represent the stress and velocity fields over the surface of the SDH, respectively, $n_{j}\left(x\right)$ is the unit component of the outward normal to the SDH, and $S_{f}$ is the surface of the SDH. In the above expressions, *m = a* and *b* denote that the flaw is present and absent, respectively, and x is a general point on the surface of the flaw.

According to the research by Thompson and Gray, when the incident wave fields are assumed as quasi-plane waves over the surface of the SDH [19], Equation (1) can be simplified to

(2) $$V_{0}\left(\omega \right)=\beta \left(\omega \right)\left[\frac{4\pi \rho_{2}c_{\gamma 2}}{-ik_{\gamma 2}S_{R}\rho_{1}c_{p1}}\right]\int_{S_{f}}V_{T}^{\gamma }\left(x,\omega \right)V_{R}^{\gamma }\left(x,\omega \right)A\left(x,\omega \right)\exp\left(-ik_{\gamma 2}e_{s}^{\gamma }\cdot x\right)dS$$

where $\gamma \left(\gamma =p,s\right)$ represents the wave type (*p* is the longitudinal wave and *s* is the vertically polarized shear wave), $\rho_{2}$ and $c_{\gamma 2}$ are density and wave velocity in the sample, respectively, $k_{\gamma 2}$ is the corresponding wave number, $V_{T}^{\gamma }\left(x,\omega \right)$ and $V_{R}^{\gamma }\left(x,\omega \right)$ represent the velocity amplitude over the surface of the SDH radiated by the transmitter and the receiver, respectively, $e_{s}^{\gamma }$ is the unit vector in the scattering direction, and $A\left(x,\omega \right)$ is the flaw scattering amplitude quantity. 

When the pulse-echo method is used, we have $V_{T}^{\gamma }\left(x,\omega \right)=V_{R}^{\gamma }\left(x,\omega \right)$. If the radius of the SDH is small, the variations of the incident wave beam can be negligible over the SDH cross-section. Thus, Equation (2) is further reduced to

(3) $$V_{0}\left(\omega \right)=\beta \left(\omega \right)\left[\frac{4\pi \rho_{2}c_{\gamma 2}}{-ik_{\gamma 2}S_{R}\rho_{1}c_{p1}}\right]\int_{C}Α\left(x,\omega \right)\exp\left(-ik_{\gamma 2}e_{s}^{\gamma }\cdot x\right)dc{\int_{L}\left(V_{T}^{\gamma }\left(x,\omega \right)\right)}^{2}dl$$

where *C* represents the curve integral path along the circumferential direction of the SDH, and *L* represents the integral path along the axial direction of the SDH. By introducing Kirchhoff approximation for flaw far-field scattering amplitude, the integration for the SDH scattering component can be written as

(4) $$F\left(\omega \right)=L\int_{C}Α\left(x,\omega \right)\exp\left(-ik_{\gamma 2}e_{s}^{\gamma }\cdot x\right)dc=\frac{\left(k_{\gamma 2}b\right)L}{2}\left[J_{1}\left(2k_{\gamma 2}b\right)-iS_{1}\left(2k_{\gamma 2}b\right)\right]+\frac{i\left(k_{\gamma 2}b\right)L}{\pi }$$

where $J_{1}$ and $S_{1}$ are the first-order Bessel function and Struve function, respectively, and b is the radius of the SDH. 

Substituting Equation (4) into Equation (3), one can obtain the ultrasonic measurement model for predicting the responses of the SDH in pulse-echo mode as 

(5) $$V_{0}\left(\omega \right)=\beta \left(\omega \right)\left[\frac{4\pi \rho_{2}c_{\gamma 2}}{-ik_{\gamma 2}S_{R}\rho_{1}c_{p1}}\right]\frac{F\left(\omega \right)}{L}{\int_{L}\left(V_{T}^{\gamma }\left(x,\omega \right)\right)}^{2}dl.\;$$

It can be seen from Equation (5) that the UMM for predicting signals from an SDH contains the system function, the ultrasonic wave beam model, and the flaw scattering model. In order to predict the flaw responses, we still need to calibrate the inspection system function using a line-focused transducer and calculate the wave beams over the flaw surface radiated by the line-focused transducer. These two parts will be discussed in the following sections.

### 2.2. MGB Model for Wave Beam Calculation

Here, we will introduce the MGB model to calculate the wave beams in two-layer media radiated by a line-focused transducer. When a uniformly distributed circular planar piston transducer is used, the sound source can be expressed as

(6) $$p_{0}\left(x^{\prime },y^{\prime },0\right)=\left\{\begin{array}{c} p_{0}\begin{array}{cc} & {x^{\prime }}^{2}+{y^{\prime }}^{2}\leq a^{2} \end{array}\\ 0\begin{array}{cc} & {x^{\prime }}^{2}+{y^{\prime }}^{2}>a^{2} \end{array} \end{array}\right.$$

where $p_{0}$ is the initial sound pressure on the transducer surface, and a is the radius of the circular transducer. According to Wen and Breazeale [20], the uniformly distributed sound pressure on the surface of the transducer can be expressed using a superposition of a set of Gaussian beams as

(7) $$p_{0}\left(x^{\prime },y^{\prime },0\right)=\sum_{m=1}^{25}p_{0}A_{m}\exp\left(-B_{xm}{x^{\prime }}^{2}/a^{2}-B_{ym}{y^{\prime }}^{2}/a^{2}\right)$$

where ${Α}_{m}$ and $B_{xm}=B_{ym}=B_{m}$ are 25 groups of complex coefficients [21]. When a focused transducer is used, we can introduce a phase correction term to account for the effects of the sound source curvature. Specially, when a line-focused transducer is used, the phase term can be added in the corresponding focal plane. In our measurement case, the beam will focus on the y-axis, and then we can write the Gaussian coefficient as [22]

(8) $$B_{xm}=B_{m}+\frac{ik_{p1}a^{2}}{2f_{t}};\;B_{ym}=B_{m}$$

where $k_{p1}$ is the wave number in the water, and $f_{t}$ is the focal length of the line-focused transducer.

The wave beam fields radiated by such a transducer can be modeled by combining the MGB sound source and the Green’s function. In addition, the ABCD matrix with interface curvature factors [23] can be introduced in the MGB model to calculate the wave fields in the immersion curved component. In a general case, when the line-focused transducer radiates the sound waves in two-layer media, the wave velocity field in the second layer of media can be expressed as

(9) $$\begin{array}{l} V_{T}^{\gamma }\left(x,\omega \right)=\sum_{m=1}^{25}T_{12}^{\gamma ;p}{\left[V_{1}^{p}\left(0\right)\right]}_{m}\frac{\sqrt{\det{\left[M_{2}^{\gamma }\left(z_{2}\right)\right]}_{m}}}{\sqrt{\det{\left[M_{2}^{\gamma }\left(0\right)\right]}_{m}}}\frac{\sqrt{\det{\left[M_{1}^{p}\left(z_{1}\right)\right]}_{m}}}{\sqrt{\det{\left[M_{1}^{p}\left(0\right)\right]}_{m}}}\\ \cdot \exp\left[\left(i\frac{\omega }{c_{p1}}\right)z_{1}+\left(i\frac{\omega }{c_{\gamma 2}}\right)z_{2}+i\frac{\omega }{2c_{p1}}y^{T}{\left[c_{p1}M_{2}^{\gamma }\left(z_{2}\right)\right]}_{m}y-\alpha_{p1}z_{1}-\alpha_{\gamma 2}z_{2}\right] \end{array}$$

where $T_{12}^{\gamma ;p}$ is the transmission coefficient when the incident longitudinal wave in water is converted to wave type of γ in the sample, and the detailed expression can be found in Reference [9]. $z_{r}\left(r=1,2\right)$ is the wave propagation path in the *r*-th layer medium, $y^{T}=\left(x_{2},y_{2}\right)$, and $\alpha_{\gamma r}$ is the attenuation coefficient of wave type of γ in the *r*-th layer medium. ${\left[V_{1}^{p}\left(0\right)\right]}_{m}$ is the initial sound pressure over the transducer surface, and ${\left[M_{1}^{p}\left(0\right)\right]}_{m}$ is the initial matrix, which are expressed as

(10) $${\left[V_{1}^{p}\left(0\right)\right]}_{m}={Α}_{m}v_{0}\left(\omega \right)\;\mathrm{and}\;$$

(11) $${\left[M_{1}^{p}\left(0\right)\right]}_{m}=\left[\begin{array}{cc} \frac{iB_{xm}}{c_{p1}D_{R}} & 0\\ 0 & \frac{iB_{ym}}{c_{p1}D_{R}} \end{array}\right]$$

where $v_{0}=p_{0}/\rho_{1}c_{1}$ is the initial wave velocity field, and $D_{R}=k_{p1}a^{2}/2$ is the Rayleigh distance in water. 

In Equation (9), $M_{1}^{p}\left(0\right)$, $M_{1}^{p}\left(z_{1}\right)$, $M_{2}^{\gamma }\left(0\right)$, and $M_{2}^{\gamma }\left(z_{2}\right)$ are all 2  ×  2 matrices, and they obey the following transfer rules:

(12) $$M_{m}^{\gamma_{m}}\left(P_{j+1}\right)=\left[D_{r}^{d}M_{m}^{\gamma_{m}}\left(P_{j}\right)+C_{r}^{d}\right]{\left[{Β}_{r}^{d}M_{m}^{\gamma_{m}}\left(P_{j}\right)+{Α}_{r}^{d}\right]}^{-1}\;\mathrm{and}\;$$

(13) $$M_{m+1}^{\gamma_{m+1}}\left(P_{j}\right)=\left[D_{r}^{t}M_{m}^{\gamma_{m}}\left(P_{j}\right)+C_{r}^{t}\right]{\left[{Β}_{r}^{t}M_{m}^{\gamma_{m}}\left(P_{j}\right)+{Α}_{r}^{t}\right]}^{-1}$$

where $P_{j}$ and $P_{j+1}$ are the two different positions at the propagation path in the medium. When the wave beam propagates in the *r*-th layer, the propagation transfer matrices ${Α}_{r}^{d}$, $B_{r}^{d}$, $C_{r}^{d}$ and $D_{r}^{d}$ are expressed as follows:

(14) $${Α}_{r}^{d}=D_{r}^{d}=I,\;B_{r}^{d}=c_{\gamma r}z_{r}I,\;C_{r}^{d}=O$$

where I is the 2×2 unit matrix, and O is the 2×2 zero matrix. 

When the wave propagates from the first layer to the second layer, the transmission transfer matrices ${Α}_{r}^{t}$, $B_{r}^{t}$, $C_{r}^{t}$ and $D_{r}^{t}$ are expressed as

(15) $${Α}_{r}^{t}=\left[\begin{array}{cc} \frac{\cos\theta_{\gamma 2}}{\cos\theta_{p1}} & 0\\ 0 & 1 \end{array}\right],\;B_{r}^{t}=O,\;C_{r}^{t}=\left(\frac{\cos\theta_{p1}}{c_{p1}}-\frac{\cos\theta_{\gamma 2}}{c_{\gamma 2}}\right)\left[\begin{array}{cc} \frac{h_{11}}{\cos\theta_{\gamma 2}\cos\theta_{p1}} & \frac{h_{12}}{\cos\theta_{\gamma 2}}\\ \frac{h_{21}}{\cos\theta_{p1}} & h_{22} \end{array}\right],\;D_{r}^{t}=\left[\begin{array}{cc} \frac{\cos\theta_{p1}}{\cos\theta_{\gamma 2}} & 0\\ 0 & 1 \end{array}\right]$$

where $\theta_{p1}$ and $\theta_{\gamma 2}$ are the incident angle in water and the refraction angle in the sample, respectively. *h* is the curvature-affected parameter, and when the axis of the cylinder component is along the y-axis, we have $h_{11}=1/R$, and $h_{12}=h_{21}=h_{22}=0$. When all the parameters in the measurement system are known, through Equation (9), the wave beam over the surface of the SDH in a cylinder component can be calculated with computational efficiency. 

### 2.3. System Function Determination

The system function is used to describe the effects of the pulser/receiver, cabling, and transducer on the measured signal. In practical applications, the system influence factor is usually obtained by a direct measurement in a calibration setup, where the pulse-echo wave from a front planar surface at normal incidence is measured. It can be calculated by the deconvolution between the received voltage response, $V_{R}\left(\omega \right)$, from the surface and the corresponding transfer function, $t_{A}\left(\omega \right)$, as [24]

(16) $$\beta \left(\omega \right)=\frac{V_{R}\left(\omega \right)}{t_{A}\left(\omega \right)}.\;$$

To reduce the sensitivity of the deconvolution to noise, a Wiener filter can be used to obtain the system function:

(17) $$\beta \left(\omega \right)=\frac{V_{R}\left(\omega \right)t_{A}^{*}\left(\omega \right)}{{\left|t_{A}\left(\omega \right)\right|}^{2}+\varepsilon^{2}\max\left\{{\left|t_{A}\left(\omega \right)\right|}^{2}\right\}}$$

where ε is a constant that is used to represent the noise level present, and ${\left(\right)}^{*}$ indicates the complex-conjugate. $t_{A}\left(\omega \right)$ here is the acoustic/elastic transfer function for line-focused transducers, which should account for the effects of reflection coefficient, attenuation, diffraction, and curved interface, and can be derived as

(18) $$t_{A}\left(\omega \right)=2\frac{1}{S_{R}}\int_{S_{R}}V_{R}^{p}\left(x,\omega \right)dS_{R}$$

where $V_{R}^{p}\left(x,\omega \right)$ is the wave beam field reflected from the water/sample interface. It can be calculated using the equation similar to Equation (9), but the reflection coefficient $R_{12}=\left(\rho_{2}c_{p2}-\rho_{1}c_{p1}\right)/\left(\rho_{2}c_{p2}+\rho_{1}c_{p1}\right)$ is used to replace the transmission coefficient, the propagation distance z_2_ is replaced by z_1_, and in Equation (15), $c_{\gamma 2}$ is replaced by $c_{p1}$ and $\cos\theta_{\gamma 2}$ is replaced by $\cos\theta_{p1}$. Note that the phase correction terms for a line-focused receiver can be employed to simplify the calculation process. 

Since the MGB model-based transfer function for calibration setup works well at different distances, it will be shown that the calibration process can be conducted at a wide range of measurement distances, eliminating the limitation of using the signal reflected from the focal plane. In addition, the effects of curvatures on the wave beams are taken into account, thus, it will be shown that the system function can also be calibrated through curved interfaces. 

## 3. Numerical Results for the Propagation Wave

The wave beams radiated by a line-focused transducer and propagating in the water–aluminum media were simulated. It should be stressed that when a large component, such as a vehicle axis or a pipeline is tested using the ultrasonic method, the driving frequency lower than 20 MHz is usually used because the wave at a higher frequency will attenuate severely. In addition, in order to ensure the adequate detection resolution, a frequency larger than 1 MHz should be selected. In the simulations, the driving frequency was selected as 10 MHz. The line-focused transducer has a diameter of 12.7 mm and focal length of 55.9 mm. The density and longitudinal wave velocity of water were $\rho_{1}$ = 1000 kg/m^3^ and $c_{p1}$ = 1485 m/s, respectively. The density of the aluminum sample was $\rho_{2}$ = 2777 kg/m^3^, and the longitudinal and shear wave velocities were 6384 m/s and 3139.7 m/s, respectively. Attenuation was neglected in the simulations. The initial particle velocity was selected as 1 m/s. The propagation and refraction wave beams can be calculated using Equation (9) when all the parameters are known. 

**Figure 2:**
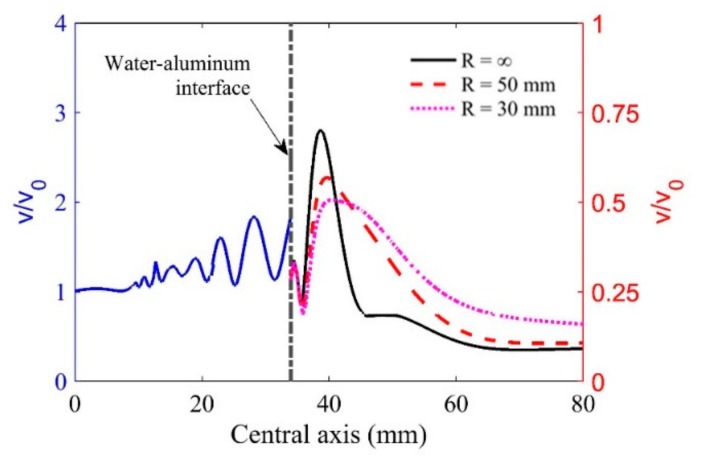
Particle velocities on the central axis in water–aluminum media at normal incidence.

Figure 2 shows the on-axis wave particle velocities in the two-layer media when the wave incidents the interface normally. The water path was selected as 34 mm, and the radii of curvature R = ∞ (plane), 50 mm, and 30 mm were used for investigating their effects on the wave beam distributions. It is observed from Figure 2 that the maximum wave particle velocity at the focal position decreases as curvature increases due to the defocusing performance of the convex interface. In addition, the curvatures will change the focal length significantly. Therefore, the curvature effects must be considered when we calculate the focal position in cylinder components. It should also be noted that the focused wave beams may lose accuracy when the radius of curvature is very small because the paraxial approximation can fail under this condition. Thus, the model only works well when the radii of cylindrical components are larger than the radius of the transducer. 

**Figure 3:**
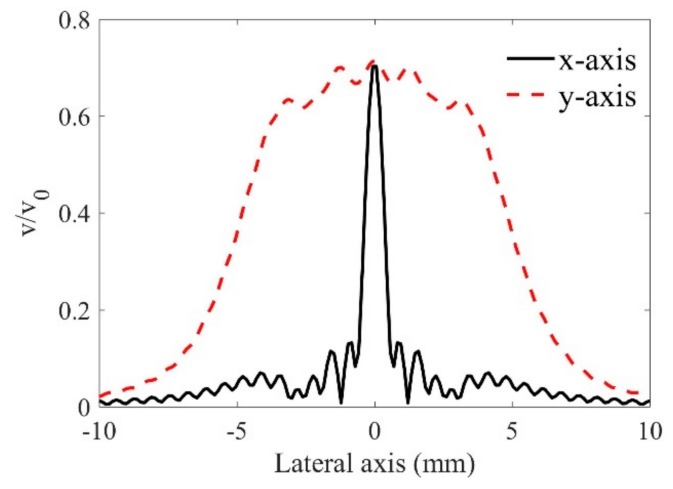
Particle velocities on the lateral axis at the focal position.

The lateral-axis velocity results at the actual focal position are shown in Figure 3. Since the wave beams are not centrosymmetric, both the results in the x- and y-axis are plotted (the coordinate is shown in Figure 1). Although the curvatures may change the beam focusing behaviors, here only the results at the case of R = ∞ are shown for the purpose of discussion. One can find the basic wave beam behaviors of the line-focused transducer from Figure 3: The main energy is concentrated at the y-axis, while at the x-axis, the energy deceases quickly when it goes far from the center axis. This non-centrosymmetric behavior is much different from that of a point-focused transducer, whose wave beam profiles are well discussed in other works [9].

**Figure 4:**
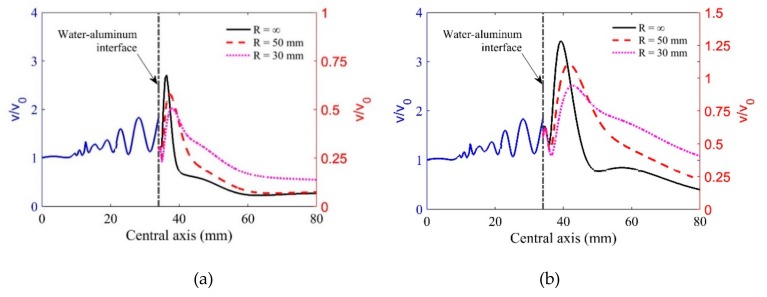
Particle velocities on the central axis in water–aluminum media: (**a**) longitudinal wave at the incidence angle of 10° and (**b**) shear wave at the incidence angle of 20°.

Figure 4a,b show the on-axis velocity results for the longitudinal wave at the incidence angle of 10° and shear wave at the incidence angle of 20°, respectively. It can be seen that both the longitudinal and shear wave beams show good focusing behaviors. Due to the changing transmission coefficients, the peak values of longitudinal wave particle velocities at oblique incidence are smaller than those in normal incidence. By comparison, it was found that the move-converted shear wave has the largest peak values of wave particle velocities because of the small impendence difference. In addition, the converted shear waves show a longer focal length since the Rayleigh distance for the shear wave is longer than the one for the longitudinal wave in aluminum. The simulation results will be helpful to design the testing program and will be used for explaining the detection results in the following section. 

## 4. Experimental Results and Discussions

The schematic diagram of the experimental test is shown in Figure 1. A line-focused transducer (Olympus V308, central frequency of 10 MHz, Tokyo, Japan) was used in the experiment. The effective focal length and radius were calibrated by using our proposed method [22], and these values have been shown in Section 3. The transducer was supported in water by a manipulator that has three degrees of freedom in movement and two in rotation. A pulser/receiver (DPR300, JSR, Rochester, NY, USA) was used to drive the line-focused transducer and receive the pulse-echo signals. Electrical output signals were received by an ultrasonic card (Ultratek PCIUT3100, Walnut Creek, CA) and processed with an industrial computer. 

LY12 aluminum alloy samples with three different shapes were designed. One was a cuboid with a size of 50 × 40 × 20 mm^3^, and the other two were semicircular cylinders, which had a thickness of 40 mm, and radii of 30 and 50 mm (shown in Figure 1), respectively. An SDH with a radius of 0.5 mm was manufactured at the metal depth of 5 mm in each sample. The attenuation coefficients in the sample were measured as $\alpha_{p2}=27\times 10^{-15}f^{2}$ Np/m and $\alpha_{s2}=53\times 10^{-15}f^{2}$ Np/m (f in Hz) for longitudinal and shear waves, respectively. The attenuation coefficient of water was $\alpha_{p1}=25.3\times 10^{-15}f^{2}$ Np/m. 

The system function with the line-focused transducer was calibrated first. By changing the incident angles, the signals from the SDHs were then predicted and measured using both longitudinal and shear waves. The detailed results are shown in the following sections. 

### 4.1. Calibrated System Function

**Figure 5:**
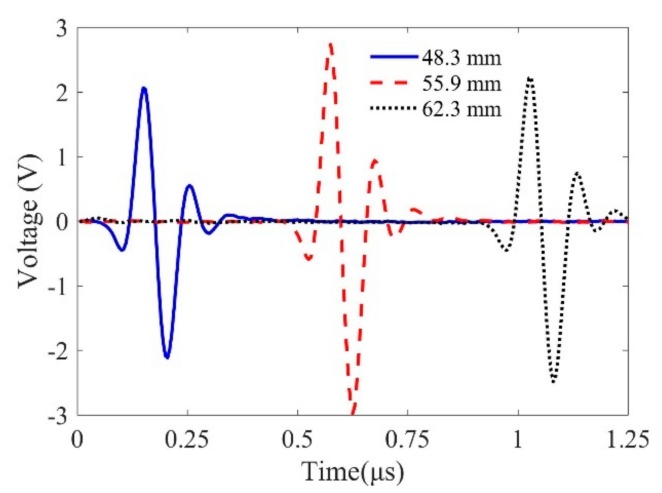
Reference signals reflected from the front surface for calibrating the system function. Time is shifted for comparison.

**Figure 6:**
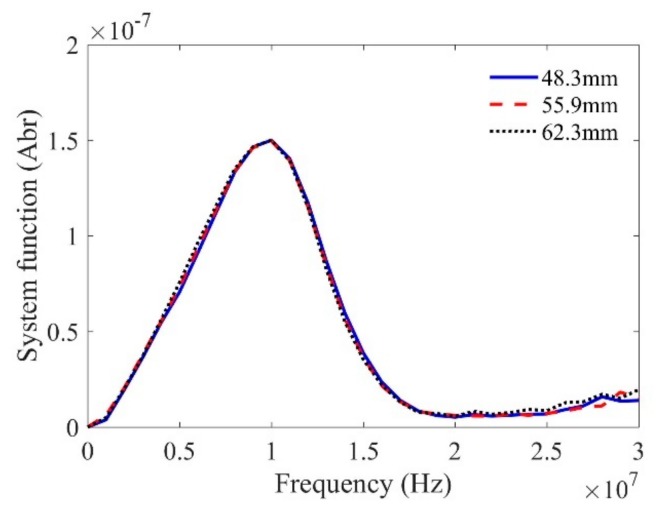
Calibrated system function using the above measured reference signals in Figure 5.

The system function is calibrated using a planar sample first. The sensitivity of the Wiener filter parameter is selected as 0.05, which can filter out most “noise” in a general ultrasonic measurement system. Although the magnitudes of the measured reference signals in Figure 5 are different due to the diffraction of the focused beams, the calibrated system functions in Figure 6 show good agreement. Therefore, these results demonstrate the effectiveness of the proposed method to calibrate the system function for a line-focused transducer in a wide range of distances. 

**Figure 7:**
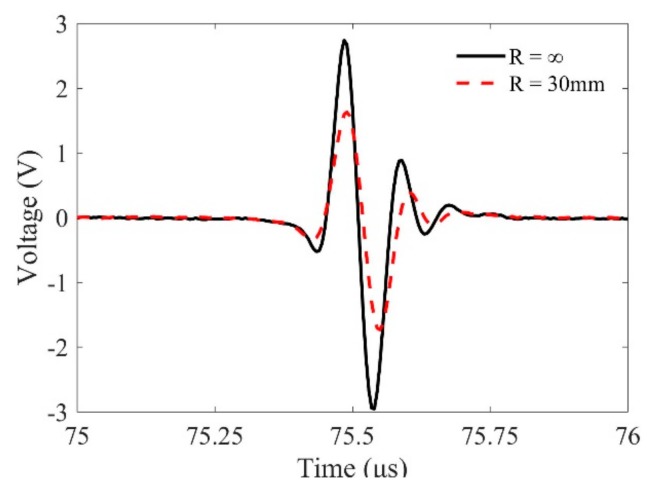
Reference signals reflected from planar and curved front surfaces for calibrating the system function.

**Figure 8:**
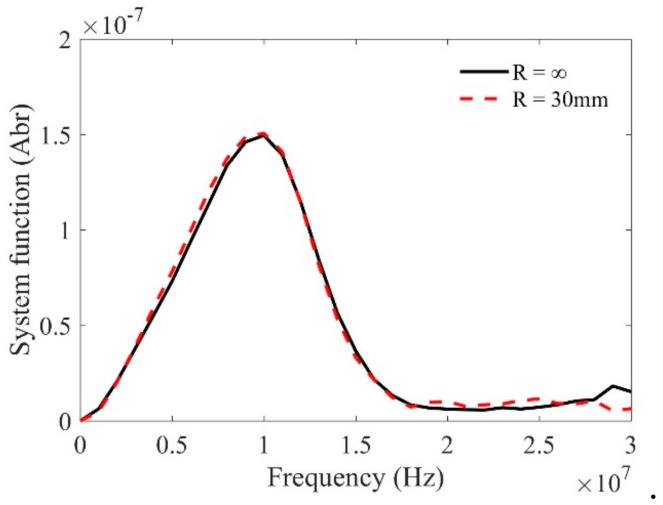
Calibrated system function using the above measured reference signals in Figure 7.

We then check the calibration method for the system function using curved interfaces. Figure 7 shows the pulse-echo wave signals reflected from a planar interface and a cylindrical interface with curvature of 30 mm when the water path is selected as 55.9 mm. One can find that due to the effects of curvatures, the magnitudes, wave forms, and even the phase properties of these measured signals are slightly different. However, because the effects of the curvatures are taken into account in the transfer function, the calibrated system functions in Figure 8 show good agreement. The agreement verifies the effectiveness of the calibration method using reference signals reflected from a curved interface. On the contrary, by checking the calibrated system function, one can judge that the transducer focusing line is parallel to the generatrix of the cylinder.

One may observe that variation of the calibration system functions also exists, especially at high frequency. It may be a result of the precision of the equipment and the electrical instruments or variability in the construction of the focused transducer. It should also be noted that errors may exist in the calibration transfer function because the MGB model (even the Rayleigh Integral method) does not capture the physical behavior of the focused transducer boundaries [25]. However, these errors are not significant, and in the following predictions, the system function obtained using the signal with a water path of 55.9 mm is used. 

### 4.2. Results Obtained Using Longitudinal Waves

**Figure 9:**
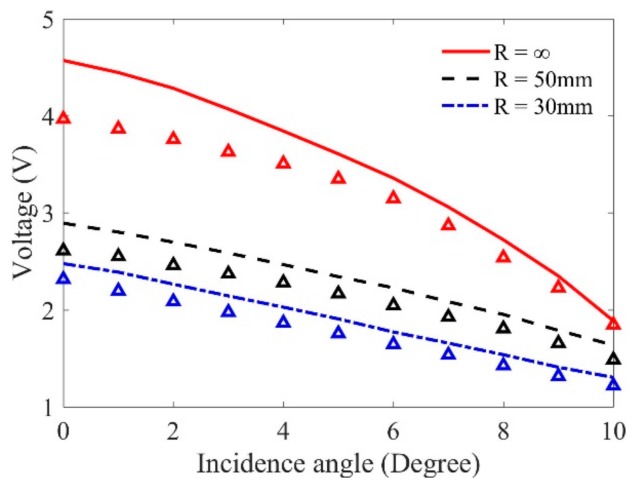
Comparison of wave magnitude results between predictions (lines) and measurements (triangles) using longitudinal waves when the curvatures and incidence angle vary.

When the system function is obtained, the wave beams over the center of the SDH are simulated, and the Kirchhoff scattering approximation for an SDH is employed. The pulse-echo signals reflected from the SDH can be predicted by using the ultrasonic measurement model, Equation (5). In these predictions, the wave beam will be focused at the center of the SDH. Here, we will focus on the results obtained using longitudinal waves. When the incident angle ($\theta_{p1}$) is given, the refraction angle ($\theta_{p2}$) of the test block can be obtained by Snell’s law. In order to focus the wave beam at the surface of the SDH, the propagation distance in the sample is calculated based on the simulation results, and the propagation in water, $z_{1}$ will be then well selected. The predicted maximum pulse-echo signals at three samples versus incident angles are shown in Figure 9. According to Snell’s law, the first critical angle of the water-–aluminum interface is determined as 13.40°. The incident angles from 0 to 10° are selected because in these conditions the SDH can be well-detected using longitudinal waves. It can be found through these simulations that with the increasing of the inclination angles and curvatures, the amplitudes of predicted flaw signals become smaller. The simulation results agree with the simulations in Section 3. 

Experiments were conducted to measure the pulse-echo flaw signals using longitudinal waves. The rotation angle of the transducer is controlled by the manipulator, and the propagation distances are adjusted by calculating the time-of-flight. Experimental results indicate that the maximum flaw signals are measured when the central ray of the propagation wave beam crosses the central axis of the SDH. The maximum amplitudes of the measured flaw responses are shown in Figure 9. We can find through the comparisons in Figure 9 that the variation tendencies of the measured results as the curvatures and incidence angles change agree with the predictions, although the measured signal magnitudes are slightly smaller than the predictions (the maximum error is within 12%). 

**Figure 10:**
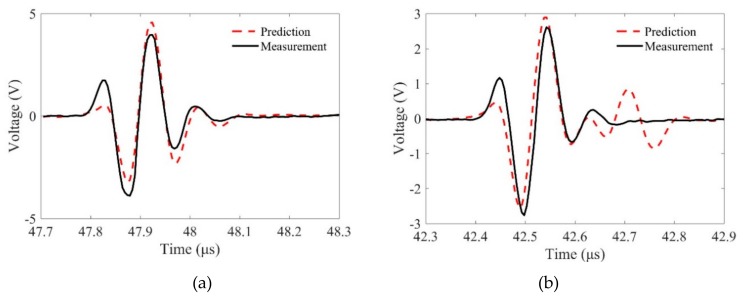

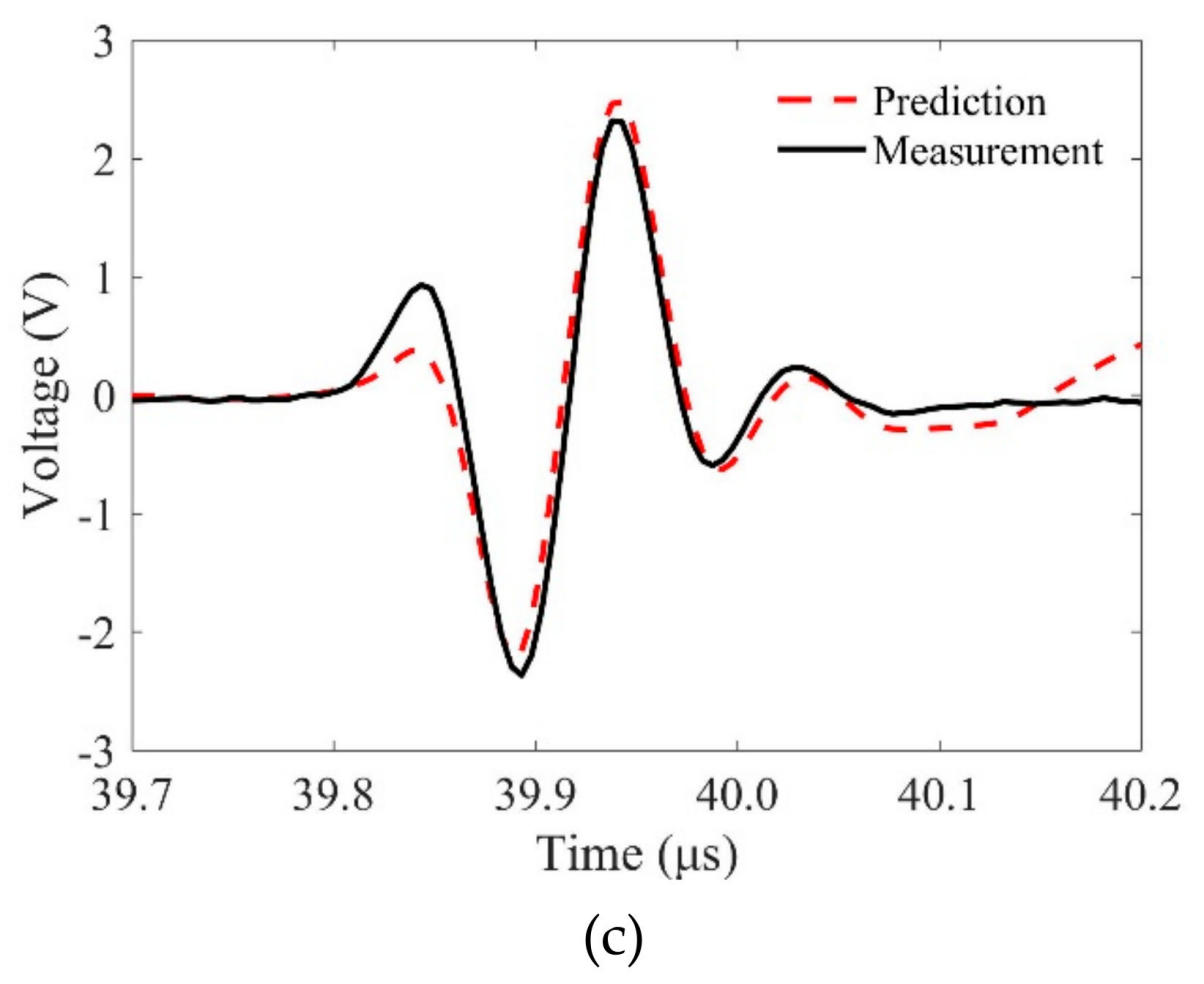
Comparisons of the flaw signals between predictions and measurements at normal incidence, $\theta_{p1}=0^{\circ }$ when the radii of curvature are (**a**) R = ∞, (**b**) R = 50 mm, and (**c**) R = 30 mm.

**Figure 11:**
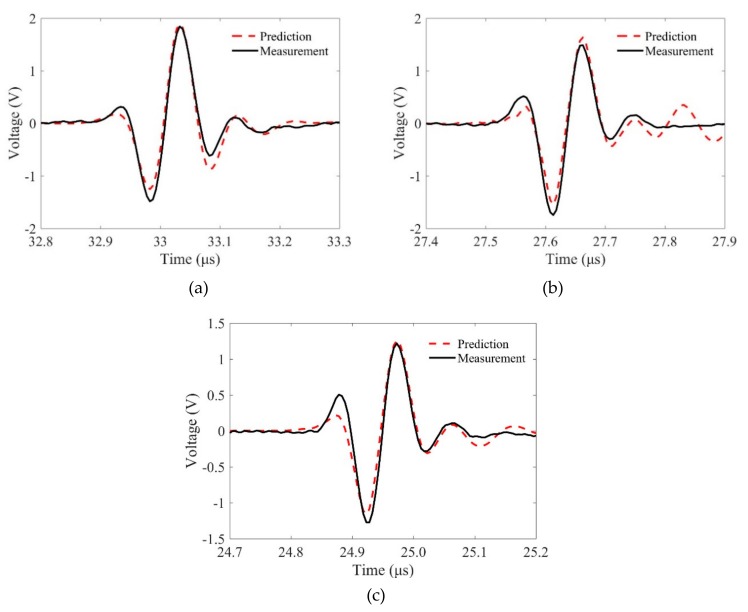
Comparisons of the flaw signals between predictions and measurements at incidence angle of $\theta_{p1}=10^{\circ }$ when the radii of curvature are (**a**) R = ∞, (**b**) R = 50 mm, and (**c**) R = 30 mm.

We then compared the predicted and measured flaw signals in detail. Figure 10 and Figure 11 show the comparison results for different samples when the incidence angles are $\theta_{p1}=0$ and $\theta_{p1}=10^{\circ }$, respectively. It is observed that the phase characteristics of these signals agree well. However, the magnitudes of the predictions and measurements show slight differences. First, these differences may come from the measurement errors. When the transducer focusing line is not parallel, the measured phase properties will be different from the predicted results and the measured flaw signals are much smaller. Second, the reason for these differences may be that the Kirchhoff approximation for the SDH scattering model only accounts for the scattering from the front surface of an SDH. The errors will decrease when $k_{p2}b$ increases [9], and we will show that in the same condition, the predictions using shear waves have smaller errors. Third, the proposed MGB model for calculating the propagation wave beams is based on the paraxial approximation, which may lose accuracy when the wave propagates in two-layer media. In general, the agreements between the predictions and measurements verify the proposed model for predicting flaw responses using longitudinal waves with line-focused transducers. 

### 4.3. Results Obtained Using Shear Waves

**Figure 12:**
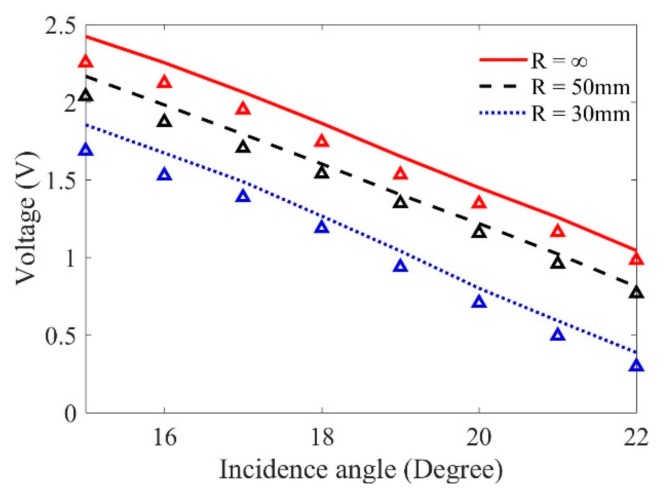
Comparison of wave magnitude results between the predictions and measurement using mode-converted shear waves when the curvatures and incidence angle vary.

In this work, the developed UMM for predicting flaw responses via wave mode conversion is helpful in analyzing the detection results and optimizing the detection technology. The flaw responses using shear wave via mode conversion can be also predicted through Equation (5). In the prediction, we focus the shear waves at the surface of the SDH. In this condition, when the incidence angle is fixed, the shear wave propagation ray distance in the sample is calculated based on the simulation results, while the propagation distance in water can be adjusted correspondingly. According to Snell’s law, the second critical angle is 28.12°. Thus, Figure 12 shows the predicted maximum flaw signals in the samples with different curvatures when the incidence angles are from 15° to 22°. Note that in this incidence angle range, only shear waves exist in the sample, and these wave beams can be focused at the center of the SDH in each sample. A similar behavior to the results predicted using longitudinal waves is observed from Figure 12: The magnitudes of these flaw responses decrease as the incidence angles and curvatures increase.

**Figure 13:**
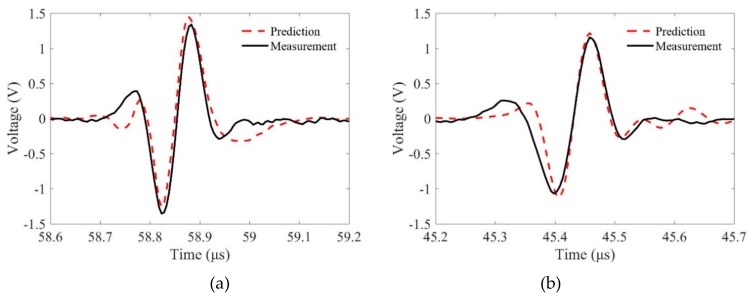

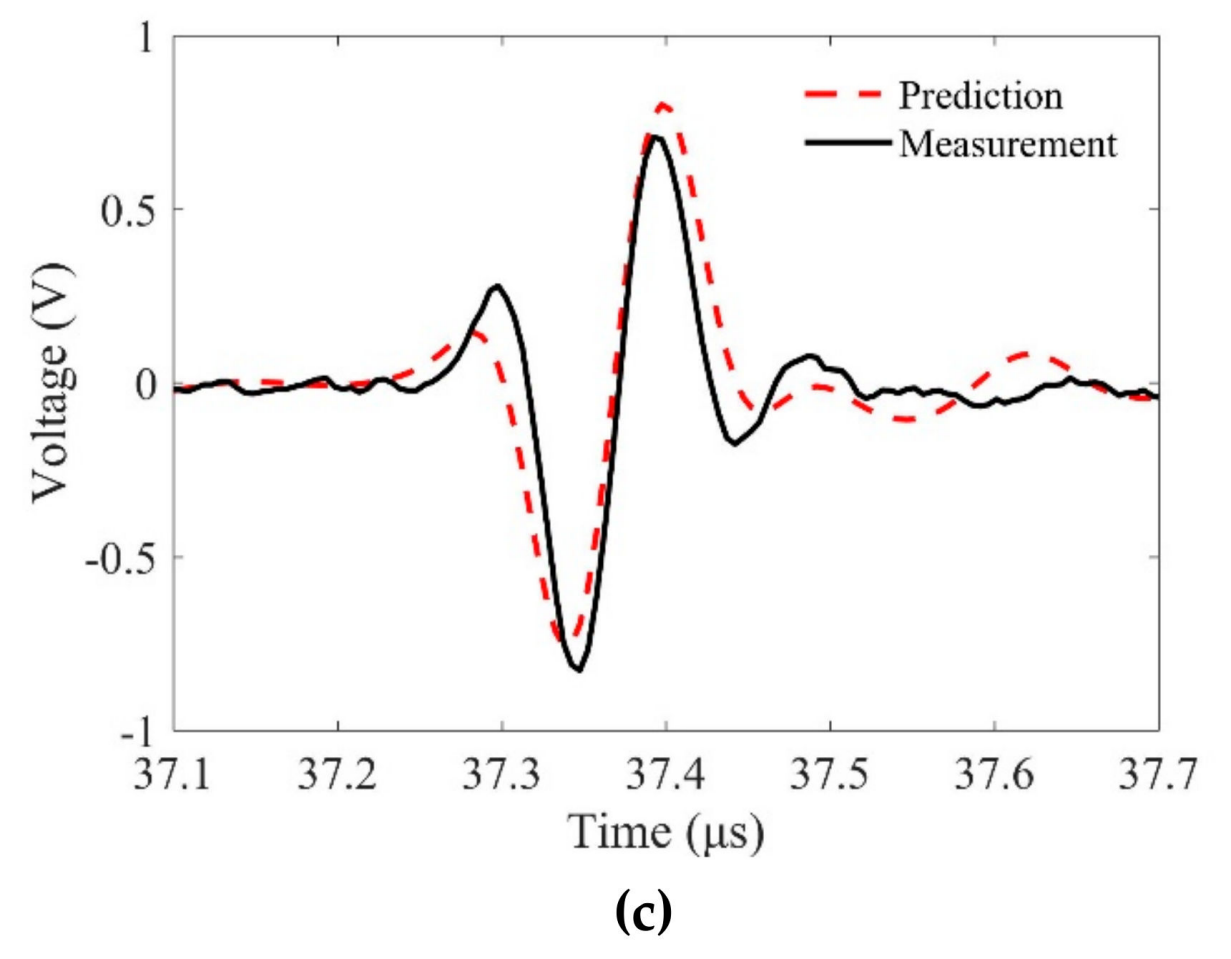
Comparisons of the flaw signals between predictions and measurements at an incidence angle of $\theta_{p1}=20^{\circ }$ when the radii of curvature are (**a**) R = ∞, (**b**) R = 50 mm, and (**c**) R = 30 mm.

Figure 13 compares the model-predicted signals and experimental measurement results of the SDHs in these samples using shear waves when the incident angle is 20°. It can be seen from these comparisons that both the wave shapes and magnitudes agree well with each other. These agreements verify the proposed model to predict flaw signals using line-focused transducers and shear waves. However, the phase properties of the predictions and measurements are slightly different. The reason may be that not all the incident angles of the radiated wave beams from a focused transducer are the same, and these behaviors are discussed in the work by Liu et al. [26]. 

## 5. Conclusions

In this work, we proposed an ultrasonic measurement model for line-focused transducers to predict flaw responses in cylindrical components. The MGB model with transfer matrices was employed to calculate the propagating wave beams in cylinder components generated by line-focused transducers. This model has high efficiency in simulating the wave beams, which can be used to help analyze wave propagation properties due to component curvatures and mode conversion radiated by a line-focused transducer. 

The system function is calibrated using reference signals and MGB model-based transfer functions. The calibrated geometrical parameters of the line-focused transducer are used to obtain the system function. Advantages of this calibration method are that the calibrated system function has a high precision and that measurement distances and the curved interfaces do not affect this calibration result. 

The flaw responses from SDHs using line-focused transducers are predicted using this model. Good agreement between the predictions and experiments validates the proposed work. The results also show that in the same measurement condition, the amplitudes of flaw signals will reduce as the curvature increases, and the flaw can be effectively detected by shear waves from mode conversion. 

Flaw responses for SDHs only are provided in this work, but the proposed method also works for other reference reflectors when the corresponding flaw scattering models are selected. Although the experiments were conducted in laboratory conditions, the proposed theory is suitable in practical applications. For example, when the component and ultrasonic test system are known, the propagating wave beams in the component can be predicted, and the program of comprehensive detection for the component can be designed based on the simulation results.

## Supplementary Materials

Supplementary data associated with simulations and experiments is uploaded for reference.
